# SGLT2 versus DPP-4 inhibitors in type 2 diabetes: a meta-analysis of outcomes

**DOI:** 10.3389/fendo.2026.1930813

**Published:** 2026-09-04

**Authors:** Xiancong Fang, Rong Li, Shengbin Liu, Xiaomin Zou, Yanling Zhong, Yalun Yang

**Affiliations:** Department of Geriatric Medicine, Huizhou Central People’s Hospital, Huizhou, Guangdong, China

**Keywords:** DPP−4 inhibitors, glycated hemoglobin, meta−analysis, SGLT2 inhibitors, type 2 diabetes mellitus

## Abstract

**Objective:**

To systematically compare sodium-glucose linked transporter 2 inhibitors (SGLT2i) and dipeptidyl peptidase 4 inhibitors (DPP4i) in glycemic control, weight reduction and genital infection risk in type 2 diabetic patients, and provide evidence-based support for individualized clinical medication.

**Materials and methods:**

A comprehensive search of PubMed, Web of Science, Cochrane Library, and EMBASE was performed for randomized controlled trials (RCTs) from database inception. Data on glycated hemoglobin, body weight, and genital infections were extracted. Risk of bias was assessed with the Cochrane ROB 2.0 tool. Meta-analyses were conducted using RevMan 5.4 and Stata 17.0, with effect sizes pooled by high- and low-dose SGLT2i subgroups. Subgroup analyses, sensitivity analyses, publication bias assessment (funnel plots plus Egger’s test), and GRADE evidence quality rating were performed.

**Results:**

10 RCTs were ultimately included. For low-dose SGLT2i, the reduction in glycated hemoglobin was not statistically different from that of DPP4i (mean difference [MD] = 0.01%, 95% confidence interval [CI]: −0.05% to 0.07%, *P* = 0.75). In contrast, high-dose SGLT2i demonstrated superior glycemic efficacy (MD = −0.15%, 95% CI: −0.27% to −0.03%, *P* = 0.01). Regardless of dosage, SGLT2i were significantly more effective than DPP4i in reducing body weight (low-dose MD = −1.69, high-dose MD = −1.92; both *P* < 0.01). Regarding safety, both low- and high-dose SGLT2i were associated with a significantly higher risk of genital infections compared with DPP4i (low-dose odds ratio [OR] = 4.25, high-dose OR = 4.03; both *P* < 0.01). Subgroup analyses suggested that the glucose-lowering effects might vary among individual SGLT2i agents, but these exploratory findings should be interpreted with caution due to the limited number of studies.

**Conclusion:**

High-dose SGLT2i offer superior glycemic control versus DPP4i; all SGLT2i doses confer significant weight loss benefits but carry a higher genital infection risk. Clinicians should therefore consider individual glycemic targets, weight status, and infection risk when selecting glucose-lowering therapies.

**Systematic Review Registration:**

https://www.crd.york.ac.uk/prospero/display_record.php?ID=CRD420261468619, identifier CRD420261468619.

## Introduction

Type 2 diabetes mellitus (T2DM) is a chronic metabolic disease with a high global prevalence. Chronic poor glycemic control can precipitate various acute and chronic complications, including cardiovascular and cerebrovascular diseases, renal injury, and retinopathy, significantly increasing patient disability rates and all-cause mortality while imposing a substantial healthcare economic burden (1, 2). With continued advances in the development of glucose-lowering agents, multiple novel oral antidiabetic drugs have been progressively introduced into clinical practice. Among these, sodium-glucose linked transporter 2 inhibitors (SGLT2i) and dipeptidyl peptidase 4 inhibitors (DPP-4i) are both recommended as second-line glucose-lowering therapies (3, 4). SGLT2i reduce renal glucose reabsorption by inhibiting the sodium-glucose linked transporter in the renal tubules, exerting glucose-lowering effects through an insulin-independent mechanism while conferring additional benefits including weight reduction, blood pressure lowering, and cardiorenal protection (5). DPP-4i delay the degradation of incretins by inhibiting dipeptidyl peptidase 4, thereby promoting insulin secretion and suppressing glucagon release in a glucose-dependent manner. These agents provide stable glycemic control with a low risk of hypoglycemia and favorable gastrointestinal tolerability (6). Given the widespread clinical application of both drug classes in T2DM management, clarifying the differences in efficacy and safety between SGLT2i and DPP-4i holds substantial practical significance for guiding individualized therapeutic selection in clinical practice.

In recent years, multiple studies have investigated the comparative efficacy and safety of SGLT2i versus DPP-4i. Wang et al. (7) conducted a meta-analysis demonstrating that SGLT2i were associated with greater reductions in glycated hemoglobin A1c (HbA1c) compared with DPP-4i, and identified heterogeneity in the glucose-lowering effect according to treatment duration and geographic region (7). Notably, that study did not include trials of subsequently marketed novel SGLT2i agents such as ertugliflozin. Maruthur et al. (8) performed a systematic review comprehensively evaluating the efficacy and safety of multiple glucose-lowering agents as monotherapy or in combination with metformin, confirming that SGLT2i and DPP-4i exhibited distinctive differences in weight control and hypoglycemia risk, with that study focused on cross-class comparisons among various antidiabetic agents (8). Natale et al. (9) conducted a systematic review in patients with diabetes and chronic kidney disease, reporting that SGLT2i significantly reduced the risks of all-cause mortality, cardiovascular death, and kidney failure. However, that study population was restricted to patients with chronic kidney disease (9).

Accordingly, the present study retrieved published randomized controlled trials (RCTs) comparing SGLT2i with DPP-4i in patients with T2DM. Meta-analyses were performed across core domains including glycemic efficacy, weight effects, and safety outcomes. Dose-stratified analyses were conducted, and sources of heterogeneity were explored. Furthermore, the GRADE (Grading of Recommendations Assessment, Development and Evaluation) framework was applied to evaluate the certainty of evidence. This study aimed to provide more comprehensive and reliable evidence-based support for individualized antidiabetic agent selection in patients with T2DM.

## Methods

### Literature screening and data extraction

#### Eligibility criteria

The inclusion criteria were as follows: (1) study design: RCTs published in English; (2) study population: patients with a confirmed diagnosis of T2DM; (3) intervention: SGLT2i administered in the experimental group (as monotherapy or in combination with a fixed background antidiabetic therapy), with DPP-4i administered in the control group (in combination with the same background therapy as the experimental group); and (4) outcome reporting: at least one of the prespecified outcome measures of this study was reported.

The exclusion criteria were as follows: (1) study type: non-RCTs, reviews, case reports, conference abstracts, letters, editorials, and other non-original studies; (2) study population: patients with type 1 diabetes mellitus or specific types of diabetes; (3) intervention: combined use of the two drug classes only, or placebo or other classes of glucose-lowering agents as the control; and (4) data availability: inability to extract complete outcome data, obvious data errors, or duplicate publications.

Following the initial screening and full-text review, two investigators independently cross-checked the included studies. For each outcome measure, studies with comparable intervention characteristics and measurement time points were grouped into the same synthesis set. For continuous outcomes (e.g., glycemic efficacy, weight change), data were separately categorized and pooled according to low and high SGLT2i dose levels. For binary safety outcomes (e.g., genital infection), dose-stratified pooling was similarly performed. Prespecified subgroup analyses were conducted by grouping studies according to stratification factors including SGLT2i type and DPP-4i type to explore sources of heterogeneity and factors influencing effect differences.

#### Information sources

The electronic databases searched included PubMed, Web of Science, the Cochrane Library, and EMBASE. The final search date for all databases was June 18, 2026. Additionally, the reference lists of all included studies and relevant systematic reviews were manually screened to supplement potentially eligible literature not captured by the database searches.

#### Search strategy

Literature searches were independently performed by two investigators. The search strategy was constructed using a combination of Medical Subject Headings (MeSH) and free-text terms. Core search terms included: “Sodium-Glucose Transporter 2 Inhibitors,” “SGLT2 Inhibitors,” “Dipeptidyl Peptidase 4 Inhibitors,” “DPP-4 Inhibitors,” “Type 2 Diabetes Mellitus,” “Randomized Controlled Trial,” and “RCT”.

Detailed information regarding the specific search strategies, search field settings, and qualification criteria for each database is presented in the **Supplementary Table** (**Supplementary Table 1**). The International Prospective Register of Systematic Reviews has our study listed under the number CRD420261468619. This systematic review and meta-analysis was conducted in strict accordance with the PRISMA 2020 statement. The entire study process followed the PRISMA-P guidelines (10).

#### Study selection process

Study selection was independently performed by two reviewers. Search results were imported into EndNote 21 for automatic duplicate detection followed by manual verification. Subsequently, titles and abstracts were independently reviewed for initial screening. Studies retained after initial screening underwent full-text review for eligibility, with reasons for exclusion documented in detail. Disagreements were resolved through discussion; in cases where consensus could not be reached, a third senior reviewer served as arbitrator.

#### Data collection process

Data extraction was independently performed by two reviewers using a predesigned standardized extraction form. Extracted data included study characteristics, baseline information, intervention details, and core outcome measures. Data entry and organization were performed using Microsoft Excel. Original study authors were not contacted for **Supplementary Data**. Extracted results were cross-checked, and discrepancies were resolved through discussion or by the adjudication of a third reviewer when agreement could not be achieved.

#### Data items

Data extracted in this study were classified into two categories: core outcome measures and study characteristic variables. All data were sourced from the full text of included studies. Content not reported in the original studies was uniformly annotated as “not reported,” and no data imputation or inference was performed.

Dose classification: For dose-stratified analyses, SGLT2 inhibitor doses were categorized as low or high as follows: empagliflozin 10 mg (low) and 25 mg (high); canagliflozin 100 mg (low) and 300 mg (high), with the intermediate 200 mg dose classified as high; ertugliflozin 5 mg (low) and 15 mg (high); dapagliflozin 5 mg (low) and 10 mg (high). For titration regimens, the final maintenance dose determined the category.

Outcome measures were defined as follows: HbA1c change was calculated as the difference between pre- and post-intervention HbA1c measurements. Mean values and standard deviations were extracted separately for low and high SGLT2i dose groups. Weight change was similarly defined as the difference between pre- and post-intervention body weight measurements, with data extracted according to dose stratification. Genital infection incidence was defined as the proportion of patients experiencing genital infections during follow-up, with the number of events and corresponding total sample size extracted for each group. Outcome data at the primary endpoint assessment time point of each study were preferentially extracted; only single time-point data were directly included if no primary endpoint time point was specified.

Study characteristic variables included basic bibliographic information, trial design, participant baseline data, and intervention protocols for each group. These variables encompassed first author, publication year, blinding method, treatment duration, follow-up duration, sample size, SGLT2i type and dosage, DPP-4i type and dosage, background therapy, as well as baseline clinical data including participant age, sex distribution, body mass index, and diabetes duration.

### Bias and evidence quality assessment

#### Risk of bias in individual studies

The risk of bias in included RCTs was assessed using the Risk-of-bias tool for randomized trials, version 2 (RoB 2.0) recommended by the Cochrane Collaboration. This tool evaluates bias across five core domains: the randomization process, deviations from intended interventions, missing outcome data, measurement of the outcome, and selection of the reported result. Risk of bias assessments for all RCTs were independently performed by two researchers trained in systematic review methodology, strictly following the standardized judgment criteria of the RoB 2.0 tool. Based on the domain-level assessments, each RCT was categorized as having low, moderate, or high overall risk of bias.

Following assessment, cross-checking was performed. For items with discrepant results, disagreements were resolved by consulting the original article and discussion; if consensus remained unattainable, the final decision was made by a third senior researcher.

#### Reporting bias

A combined qualitative and quantitative approach was employed to evaluate the risk of publication bias for each core outcome measure. Funnel plots were initially generated for each outcome to perform visual qualitative assessment. Asymmetry in the distribution of effect estimates around the pooled effect was examined to preliminarily determine whether funnel plot asymmetry existed due to unpublished negative results, small-study bias, or selective reporting. Quantitative verification was subsequently performed using Egger’s linear regression test, with statistical significance of the regression intercept (*P* < 0.05) serving as the criterion for publication bias.

#### Certainty of evidence

The certainty of evidence for each outcome measure was evaluated using the internationally adopted GRADE framework. Evidence grades for all core outcomes were independently assigned by two researchers trained in systematic review methodology. Following assessment, results were cross-checked. Discrepancies were resolved through joint discussion and re-examination of the original articles; if agreement could not be reached, the final decision was made by a third senior researcher.

### Effect measures and synthesis methods

#### Effect measures

Effect measures for data synthesis were selected according to outcome data types. For continuous outcomes (HbA1c change and weight change), the mean difference (MD) was used as the primary effect measure, with pre- to post-intervention change values uniformly employed for pooled analyses. For binary outcomes (genital infection incidence), the odds ratio (OR) was used as the primary effect measure.

For change-score standard deviations, we preferentially extracted them directly from the original reports when available. When only baseline and endpoint values with their standard deviations were reported, we calculated the change-score standard deviation using the formula: SD_change = √(SD_baseline^2^ + SD_endpoint^2^ − 2 × r × SD_baseline × SD_endpoint), assuming a conservative correlation coefficient of r = 0.5, as recommended by the Cochrane Handbook. This approach was applied consistently across all continuous outcomes. Regarding the pooling of outcomes with varying follow-up durations, we extracted data at the primary endpoint assessment time point of each study as prespecified in the original protocols. The potential impact of different follow-up lengths was subsequently examined through sensitivity analyses, rather than through *a priori* justification of a specific time window.

#### Synthesis methods

Forest plots were generated to visually display individual study results and pooled effect estimates. Meta-analyses were performed to synthesize eligible study results using RevMan 5.4 and Stata 17.0 software for statistical computations. Heterogeneity was quantified using the I^2^ statistic (I^2^ < 25% considered low heterogeneity, 25%–75% moderate heterogeneity, and > 75% high heterogeneity).

To explore potential sources of heterogeneity among study results, subgroup analyses were conducted according to prespecified stratification factors, including SGLT2i type and DPP-4i type. Two sensitivity analysis approaches were prespecified to evaluate the robustness of pooled results: (1) leave-one-out analysis, in which one study was sequentially excluded and the remaining studies re-analyzed to observe changes in pooled effect estimates, heterogeneity, and statistical significance; and (2) model switching analysis, in which fixed-effect and random-effects models were alternated to assess the influence of effect model selection on core conclusions. In addition, *post hoc* sensitivity analyses would be conducted for studies with notable clinical heterogeneity (e.g., differences in population characteristics) to further explore sources of heterogeneity and verify conclusion stability.

## Results

### Study selection results

A total of 635 records were retrieved through searches of PubMed, Web of Science, the Cochrane Library, and EMBASE (**Supplementary Figure 1**). After excluding 382 duplicate records, 253 records remained for initial screening. Following the initial screening, 187 records that did not meet the inclusion criteria were excluded, and the full texts of the remaining 66 records were obtained for further assessment. During the full-text review phase, 56 studies were sequentially excluded due to ineligible study design, mismatched study population, inconsistent intervention, missing outcome data, or duplicate publication. Ultimately, 10 studies were included in this meta-analysis.

### Characteristics of included studies

A total of 5,264 participants were enrolled across the 10 included studies. All included studies employed a double-blind design. The intervention drugs comprised four SGLT2i agents (empagliflozin, canagliflozin, ertugliflozin, and dapagliflozin), and the comparator drugs comprised two DPP-4i agents (linagliptin and sitagliptin). Each study included both low and high dose levels. The primary endpoint assessment time points were consistent with the overall follow-up durations across studies (**Table 1**). Core demographic and clinical baseline characteristics, including age, sex distribution, body mass index, glycemic parameters, renal function, blood pressure, and diabetes duration, were generally balanced between the SGLT2i and DPP-4i groups, supporting the comparability of the two groups (**Supplementary Table 2**). Only a subset of studies did not fully report secondary lipid-related parameters, which did not affect the overall baseline balance.

**Table 1:** Baseline characteristics of included studies.

First author, year	Blinding	Treatment duration (weeks)	Follow-up duration (weeks)	Primary endpoint assessment (weeks)	SGLT2i type	SGLT2i dosage	DPP-4i type	DPP-4i dosage	Background therapy
DeFronzo 2015 (11)	Double-blind	52	52	24	Empagliflozin	10 mg or 25 mg once daily	Linagliptin	5 mg once daily	Metformin
Laffel 2023 (12)	Double-blind	26	26	26	Empagliflozin	10 mg or 25 mg	Linagliptin	5 mg once daily	Metformin and/or insulin
Lavalle-González 2013 (13)	Double-blind	52	52	26	Canagliflozin	100 mg or 300 mg once daily	Sitagliptin	100 mg once daily	Metformin
Lewin 2015 (14)	Double-blind	52	52	24	Empagliflozin	10 mg or 25 mg once daily	Linagliptin	5 mg once daily	None
Pratley 2018 (15)	Double-blind	52	52	26	Ertugliflozin	5 mg or 15 mg once daily	Sitagliptin	100 mg once daily	Metformin
Roden 2013 (16)	Double-blind	24	24	24	Empagliflozin	10 mg or 25 mg once daily	Sitagliptin	100 mg once daily	None
Rosenstock 2012 (17)	Double-blind	12	12	12	Canagliflozin	100 mg, 200 mg, or 300 mg once daily	Sitagliptin	100 mg once daily	Metformin
Rosenstock 2013 (18)	Double-blind	12	12	12	Empagliflozin	10 mg or 25 mg once daily	Sitagliptin	100 mg once daily	Metformin
Schernthaner 2013 (19)	Double-blind	52	52	52	Canagliflozin	300 mg once daily	Sitagliptin	100 mg once daily	Metformin, sulfonylureas
Scott 2018 (20)	Double-blind	24	24	24	Dapagliflozin	5 mg to 10 mg once daily	Sitagliptin	100 mg once daily	Metformin, sulfonylureas

SGLT2i, sodium-glucose linked transporter 2 inhibitors; DPP-4i, dipeptidyl peptidase 4 inhibitors.

### Results of risk of bias in individual studies

Risk of bias for the 10 included RCTs was assessed using the Cochrane RoB 2.0 tool (**Supplementary Figure 2**). The assessment results indicated that the majority of studies were rated as low risk across all five domains: the randomization process, deviations from intended interventions, missing outcome data, measurement of the outcome, and selection of the reported result. Only individual studies raised some concerns in certain domains, and no study was rated as high risk. Collectively, the methodological quality of the included literature was satisfactory, supporting a high degree of confidence in the meta-analytic findings.

### Results of individual studies and synthesis results

#### HbA1c change (low-dose SGLT2i)

**Figure 1:**
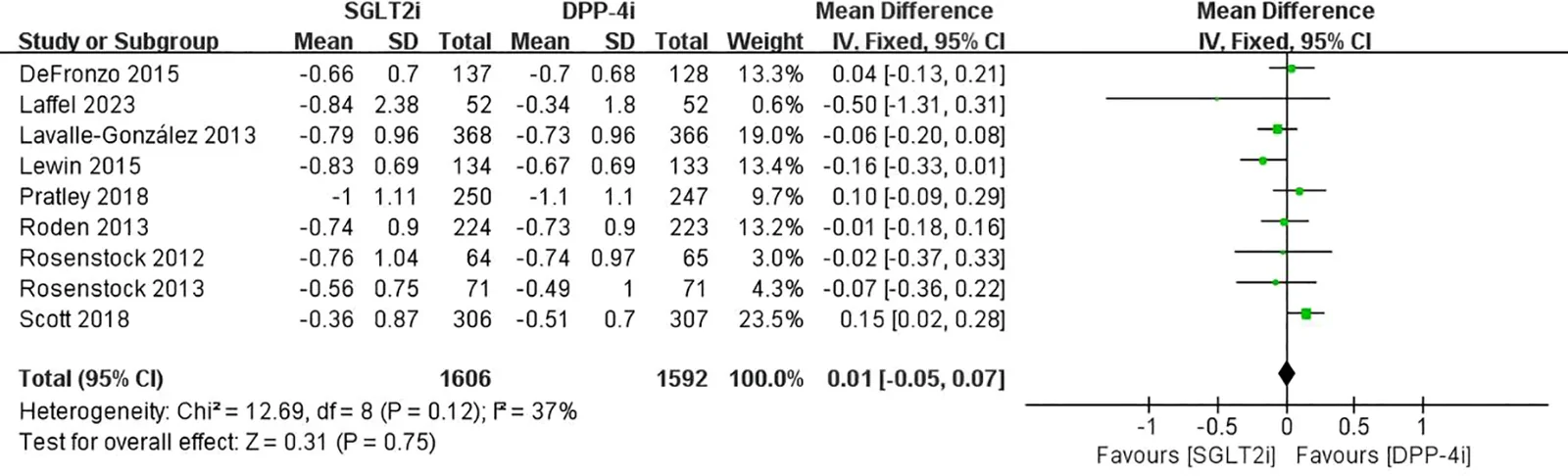
Forest plot for HbA1c (low-dose SGLT2i).

Meta-analysis was performed using a fixed-effect model. The results demonstrated no statistically significant difference between the two drug classes in HbA1c reduction, with MD = 0.01%, 95% confidence interval (CI): −0.05% to 0.07%, *Z* = 0.31, *P* = 0.75. Low-to-moderate heterogeneity was observed among studies (χ^2^ = 12.69, df = 8, *P* = 0.12, *I^2^* = 37%) (**Figure 1**). To verify the robustness of the pooled results, two sensitivity analyses were conducted: leave-one-out analysis and model switching to random-effects. Following sequential exclusion of each individual study, the pooled MD values ranged from −0.03% to 0.01%, with all 95% CIs crossing the null line and none reaching statistical significance, consistent with the primary analysis. Notably, heterogeneity was completely eliminated after excluding the study by Scott et al. (2018) (*I^2^* = 0%, *P* = 0.50), suggesting that this study was the primary contributor to heterogeneity in this outcome (20). The random-effects model yielded consistent results, with no statistically significant difference between the two drug classes in HbA1c reduction (MD = 0.00%, 95% CI: −0.08% to 0.08%, *Z* = 0.02, *P* = 0.99), confirming that the core findings were not influenced by any single study or the choice of effect model, and were therefore robust and reliable.

#### HbA1c change (high-dose SGLT2i)

**Figure 2:**
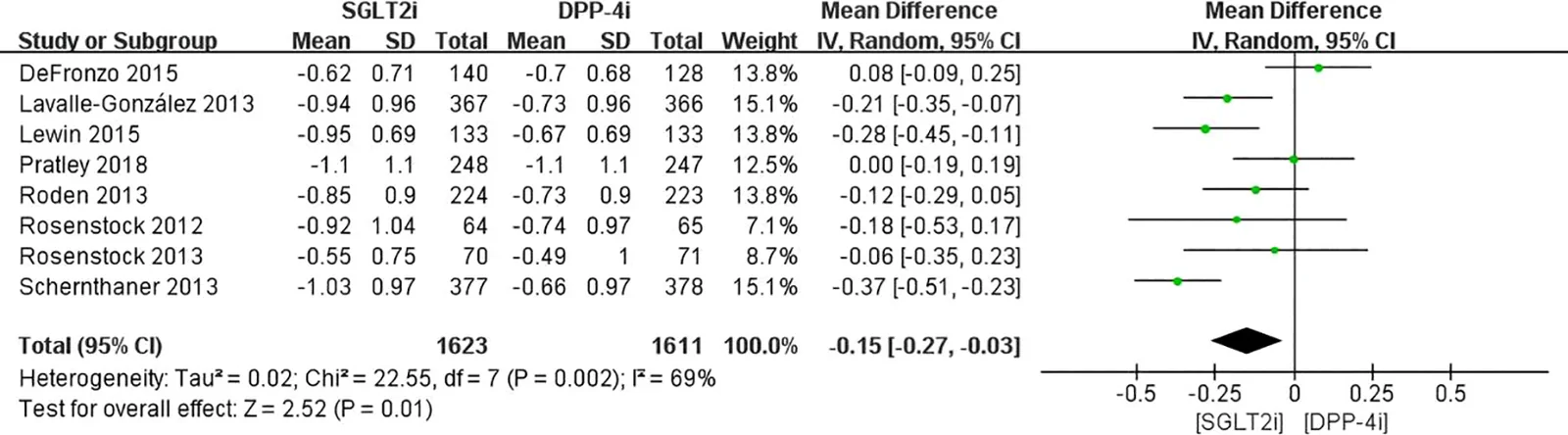
Forest plot for HbA1c (high-dose SGLT2i).

High heterogeneity was observed among studies (χ^2^ = 22.55, df = 7, *P* < 0.01, *I^2^* = 69%); consequently, a random-effects model was employed for meta-analysis. The results demonstrated that high-dose SGLT2i were significantly more effective than DPP-4i in reducing HbA1c (MD = −0.15%, 95% CI: −0.27% to −0.03%, *Z* = 2.52, *P* = 0.01) (**Figure 2**). To validate the robustness of this finding, leave-one-out sensitivity analyses were performed. Sequential exclusion of each study with re-analysis using random-effects models yielded pooled MD values ranging from −0.19 to −0.11. When the studies by Lavalle-González et al. (2013) and Lewin et al. (2015) were individually excluded, the upper limit of the 95% CI for the pooled effect reached 0, with an effect test *P*-value of 0.05 (13, 14). For all other individual exclusions, the upper limits of the 95% CIs remained below 0, and the effect test *P*-values remained < 0.05, consistently indicating superior glycemic efficacy of high-dose SGLT2i. Heterogeneity decreased to varying degrees following exclusion of any single study; notably, exclusion of the study by DeFronzo et al. (2015) reduced *I^2^* to 52% (11). Overall, the conclusions were not entirely reversed by any single study.

#### Weight change (low-dose SGLT2i)

**Figure 3:**
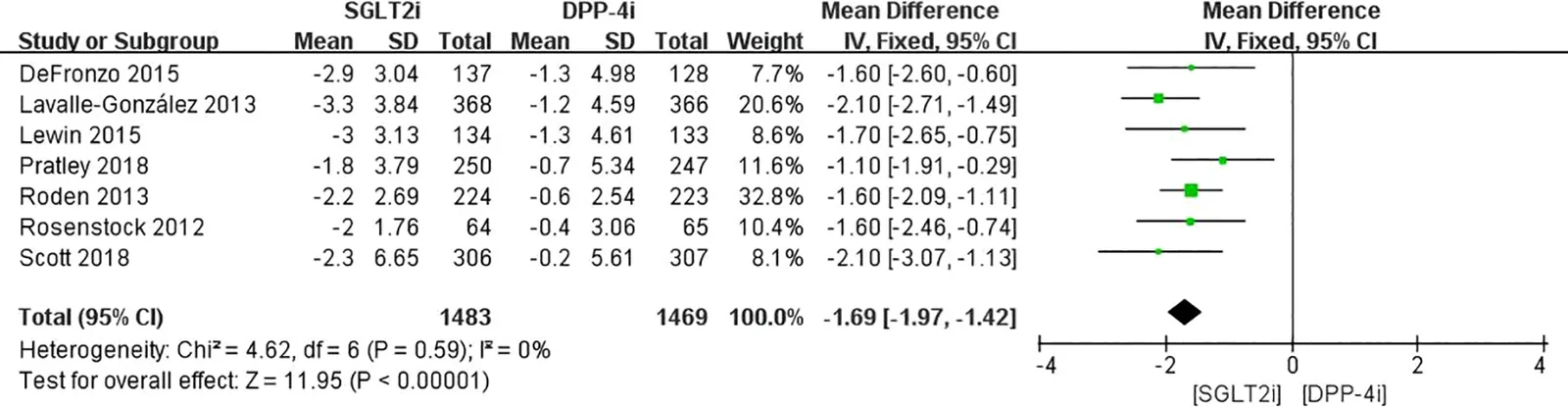
Forest plot for weight change (kg) (low-dose SGLT2i).

No statistically significant heterogeneity was observed among studies (χ^2^ = 4.62, df = 6, *P* = 0.59, *I^2^* = 0%); a fixed-effect model was therefore used for meta-analysis. The results revealed that low-dose SGLT2i were associated with significantly greater weight reduction compared with DPP-4i (MD = −1.69, 95% CI: −1.97 to −1.42, *Z* = 11.95, *P* < 0.01) (**Figure 3**). Leave-one-out sensitivity analyses using fixed-effect models yielded pooled MD values ranging from −1.77 to −1.59. Under all exclusion scenarios, the upper limits of the 95% CIs remained below 0, and the overall effect tests consistently showed *P* < 0.01. Heterogeneity remained at *I^2^* = 0% following exclusion of any single study, indicating no substantial heterogeneity and confirming the robustness and reliability of the results.

#### Weight change (high-dose SGLT2i)

**Figure 4:**
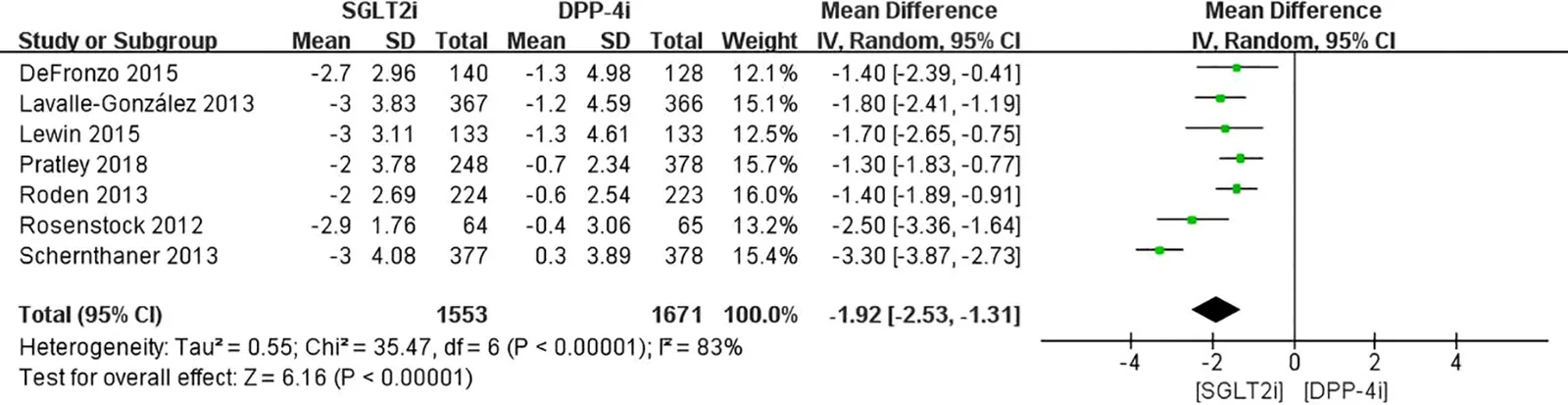
Forest plot for weight change (kg) (high-dose SGLT2i).

High heterogeneity was observed among studies (Tau^2^ = 0.55, χ^2^ = 35.47, df = 6, *P* < 0.01, *I^2^* = 83%); therefore, a random-effects model was applied for meta-analysis. The results demonstrated that high-dose SGLT2i were significantly superior to DPP-4i in weight reduction (MD = −1.92, 95% CI: −2.53 to −1.31, *Z* = 6.16, *P* < 0.01) (**Figure 4**). Leave-one-out sensitivity analyses using random-effects models yielded pooled MD values ranging from −2.03 to −1.83. Under all exclusion scenarios, the upper limits of the 95% CIs remained below 0, and the overall effect tests consistently yielded *P* < 0.01. Following exclusion of the study by Schernthaner et al. (2013), heterogeneity substantially decreased to *I^2^* = 25% (χ^2^ = 6.70, df = 5, *P* = 0.24), with a pooled MD of −1.61 (95% CI: −1.93 to −1.29, *P* < 0.01), indicating that the weight-loss benefit remained stable (19). This study had a follow-up duration of 52 weeks, whereas the remaining studies had follow-up durations of only 12, 24, or 26 weeks, suggesting that differences in follow-up duration may have contributed to the high heterogeneity observed in this group. Overall, sensitivity analyses indicated that the weight-loss advantage of high-dose SGLT2i was consistently present regardless of which individual study was excluded.

#### Genital infection (low-dose SGLT2i)

**Figure 5:**
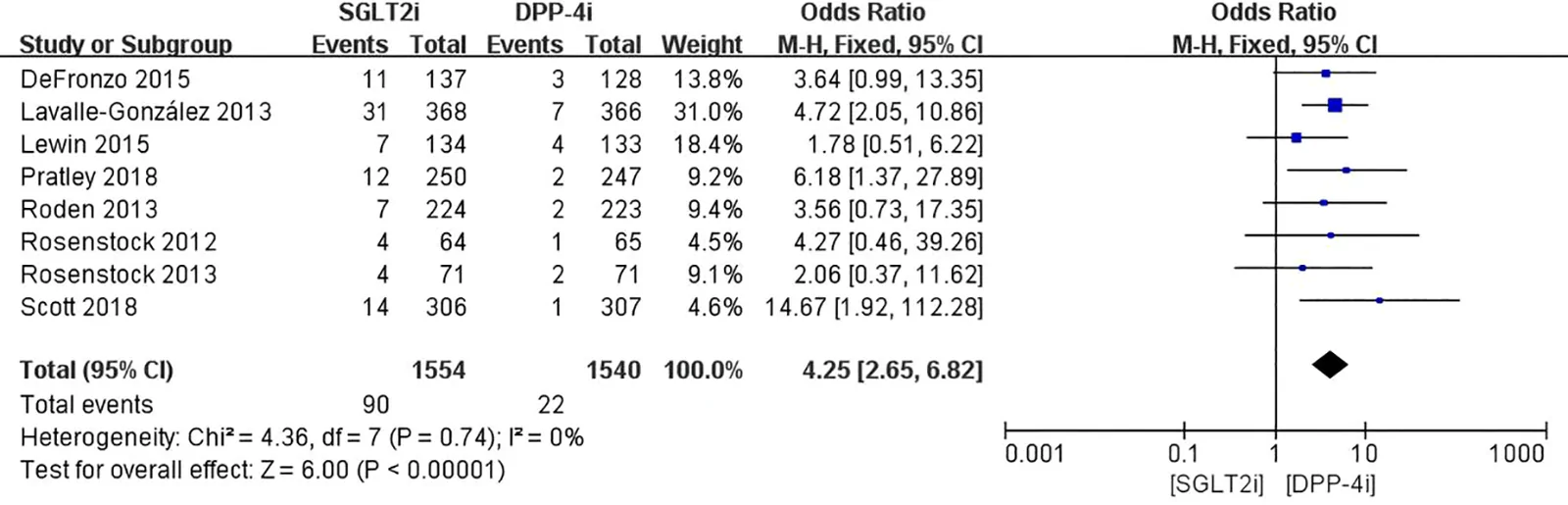
Forest plot for genital infection (low-dose SGLT2i).

No statistically significant heterogeneity was observed among studies (χ^2^ = 4.36, df = 7, *P* = 0.74, *I^2^* = 0%); a fixed-effect model was therefore used for meta-analysis. The results revealed that the SGLT2i group had a significantly higher risk of genital Infection compared with the DPP-4i group (OR = 4.25, 95% CI: 2.65 to 6.82, *Z* = 6.00, *P* < 0.01) (**Figure 5**). Leave-one-out sensitivity analyses using fixed-effect models yielded pooled OR values ranging from 3.75 to 4.81. Under all exclusion scenarios, the lower limits of the 95% CIs remained above 1, and the overall effect tests consistently showed *P* < 0.01. Furthermore, heterogeneity remained at *I^2^* = 0% following exclusion of any single study, confirming that the conclusion regarding SGLT2i-associated increased risk of genital Infection was stable and reliable regardless of which individual study was excluded.

#### Genital infection (high-dose SGLT2i)

**Figure 6:**
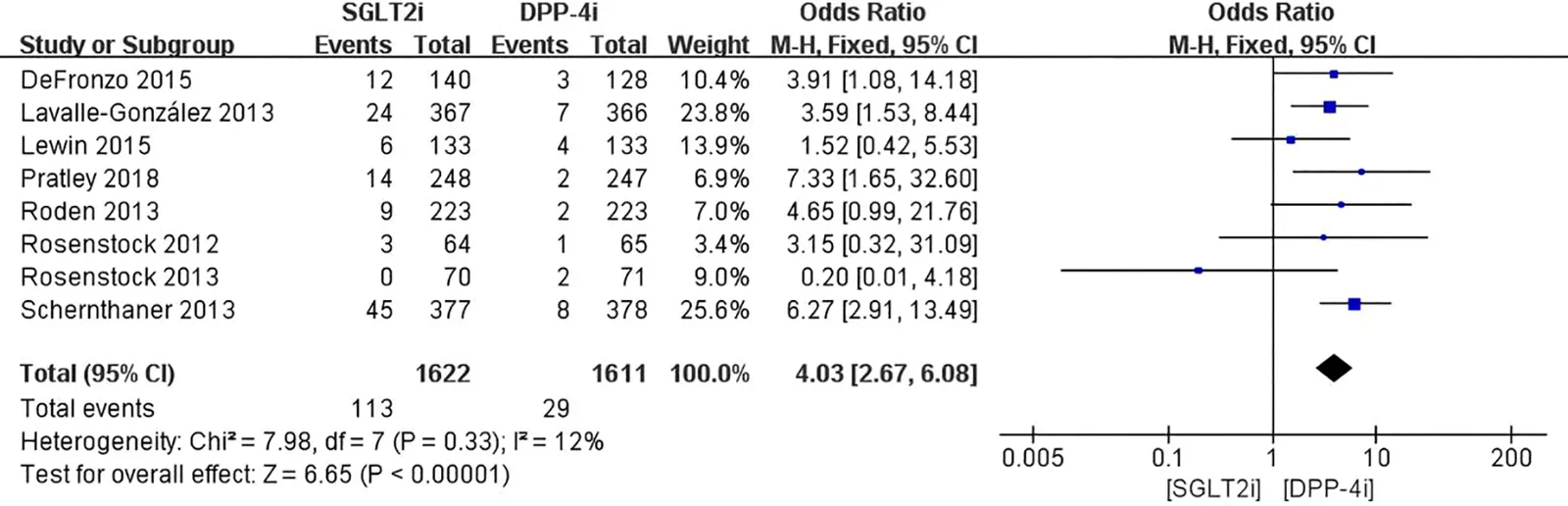
Forest plot for genital infection (high-dose SGLT2i).

Mild heterogeneity was observed among studies (χ^2^ = 7.98, df = 7, *P* = 0.33, *I^2^* = 12%); a fixed-effect model was therefore used for meta-analysis. The results demonstrated that the high-dose SGLT2i group had a significantly higher risk of genital infections compared with the DPP-4i group (OR = 4.03, 95% CI: 2.67 to 6.08, *Z* = 6.65, *P* < 0.01) (**Figure 6**). Leave-one-out sensitivity analyses using fixed-effect models yielded pooled OR values ranging from 3.26 to 4.44. Under all exclusion scenarios, the lower limits of the 95% CIs remained above 1, and the overall effect tests consistently showed *P* < 0.01. Heterogeneity remained at *I^2^* = 0%–25% following exclusion of any single study, with no substantial increase, confirming the robustness and reliability of the results regardless of which individual study was excluded.

### Subgroup analyses

Subgroup analyses were performed according to SGLT2i type. For HbA1c reduction with high-dose SGLT2i, the test for subgroup differences was statistically significant (*P* = 0.04), indicating a significant subgroup effect. Given the limited number of studies in each drug subgroup, this exploratory finding should be interpreted with caution. Among the individual agents, only canagliflozin significantly reduced HbA1c. For weight reduction, all drug subgroups demonstrated significant weight-loss benefits, with no statistically significant between-subgroup difference (*P* = 0.09). Stratification by DPP-4i type revealed that high-dose SGLT2i significantly improved HbA1c only when sitagliptin was the comparator; however, the test for subgroup differences was not significant (*P* = 0.70). High-dose SGLT2i significantly reduced body weight with both sitagliptin and linagliptin as comparators, and the between-subgroup comparison showed no significant difference in efficacy (*P* = 0.22) (**Table 2**).

**Table 2:** Subgroup analyses.

Grouping method	Outcome	Subgroup	Number of studies	Heterogeneity		Test for overall effect	
				*I* *^2^*	*P*	*Z*	*P*
Types of SGLT2i	HbA1c change (high-dose SGLT2i)	Empagliflozin	4	67%	0.03	1.17	0.24
		Canagliflozin	3	31%	0.23	4.45	<0.01
		Ertugliflozin	1	/	/	/	/
		Overall	8	69%	<0.01	2.52	0.01
		Test for subgroup differences: Chi^2^ = 6.67, df = 2 (*P* = 0.04), *I^2^* = 70.0%					
	Weight change (high-dose SGLT2i)	Empagliflozin	3	0%	0.85	7.19	<0.01
		Canagliflozin	3	84%	0.002	5.17	<0.01
		Ertugliflozin	1	/	/	/	/
		Overall	7	82%	<0.01	6.11	<0.01
		Test for subgroup differences: Chi^2^ = 4.76, df = 2 (*P* = 0.09), *I^2^* = 58.0%					
Types of DPP-4i	HbA1c change (high-dose SGLT2i)	Sitagliptin	6	57%	0.04	2.83	<0.01
		Linagliptin	2	89%	<0.01	0.56	0.58
		Overall	8	69%	<0.01	2.52	0.01
		Test for subgroup differences: Chi^2^ = 0.15, df = 1 (*P* = 0.70), *I^2^* = 0%					
	Weight change (high-dose SGLT2i)	Sitagliptin	5	87%	<0.01	14.44	<0.01
		Linagliptin	2	0%	0.67	4.46	<0.01
		Overall	7	82%	<0.01	15.06	<0.01
		Test for subgroup differences: Chi^2^ = 1.50, df = 1 (*P* = 0.22), *I^2^* = 33.4%					

HbA1c, glycated hemoglobin A1c; SGLT2i, sodium-glucose linked transporter 2 inhibitors; DPP-4i, dipeptidyl peptidase 4 inhibitors. I^2^, inconsistency index. Test for subgroup differences was used to compare the pooled effects across different subgroups.

### Results of reporting bias

Publication bias was assessed using funnel plots in conjunction with Egger’s linear regression test. The funnel plots for each outcome showed generally symmetrical distributions of study effect estimates (**Supplementary Figure 3**). Egger’s test P-values for all outcomes (HbA1c, weight, and genital infection at both low and high SGLT2i doses) were all > 0.05, indicating no statistically significant publication bias (**Supplementary Table 3**). However, given that fewer than 10 studies were included in each individual analysis, the Egger’s test has limited statistical power; therefore, these results should be interpreted with caution.

### Results of certainty of evidence

All included outcomes were derived from RCTs, with the initial evidence level set as high quality. Downgrading factors were evaluated across five domains: risk of bias, inconsistency, indirectness, imprecision, and publication bias (**Supplementary Table 4**). Overall, the methodological quality of included studies was satisfactory, with no significant downgrading due to risk of bias, indirectness, or publication bias. The certainty of evidence for most outcomes was moderate, with the exception of the low-dose SGLT2i weight reduction subgroup, which was rated as high quality. These findings suggest that the study conclusions possess good reference value.

## Discussion

### Comprehensive interpretation of results

This meta-analysis included 10 RCTs and yielded the following findings: low-dose SGLT2i showed no statistically significant difference in HbA1c reduction compared with DPP-4i; however, high-dose SGLT2i demonstrated significantly superior glycemic efficacy relative to DPP-4i. Furthermore, SGLT2i were associated with significantly greater weight reduction than DPP-4i at both low and high doses, yet conferred a significantly higher risk of genital infections.

From the glycemic efficacy perspective, the between-drug difference varied by dose level, with high-dose SGLT2i showing a greater reduction than DPP-4i while low-dose SGLT2i showed comparable effects. Low-dose SGLT2i exerted limited inhibition of renal tubular glucose reabsorption, with urinary glucose excretion not yet reaching the plateau phase; consequently, the overall glycemic reduction was comparable to that achieved by DPP-4i through the incretin pathway, resulting in no significant between-group difference in HbA1c reduction. High-dose SGLT2i further enhanced urinary glucose excretion efficiency, achieving stronger glycemic control through an insulin-independent glucose-excreting mechanism (21). In contrast, the glucose-lowering effect of DPP-4i is constrained by endogenous incretin secretion levels, exhibiting a definitive ceiling effect, such that dose escalation cannot further augment the glycemic reduction (22). However, the observed HbA1c reduction of −0.15% for high-dose SGLT2i versus DPP-4i, while statistically significant, is modest in magnitude. Given that a difference of 0.3%–0.5% is generally considered clinically meaningful for HbA1c, this modest effect suggests that the clinical advantage of high-dose SGLT2i over DPP-4i in glycemic control may be limited. Therefore, when choosing between these two drug classes, clinicians should weigh this relatively small glycemic benefit against other patient-specific factors such as weight management needs and infection risk. Ultimately, this mechanistic distinction leads to a significant divergence in glycemic efficacy between the two drug classes at high doses. Subgroup analyses suggested that the glucose-lowering potency varied to some extent across different SGLT2i agents, with canagliflozin demonstrating relatively prominent glycemic benefits. Given the limited number of studies in each drug subgroup, this exploratory finding should be interpreted with caution.

Regarding weight management, the weight-loss advantage of SGLT2i over DPP-4i was stable and significant, consistently observed across all dose strata and drug subgroups. SGLT2i induce sustained caloric loss through urinary glucose excretion, accompanied by mild natriuretic and osmotic diuretic effects, achieving gradual and stable weight reduction (23). Conversely, DPP-4i exert neutral effects on body weight, neither promoting weight loss nor inducing weight gain; therefore, the between-group difference in weight change has a clear pharmacological basis (24). The high-dose weight analysis exhibited considerable heterogeneity. Sensitivity analyses identified between-study differences in follow-up duration as the core source of heterogeneity, with the 52-week study showing substantially greater weight reduction than studies with shorter follow-up periods, suggesting that the weight-loss effect of SGLT2i accumulates over time.

In terms of safety outcomes, elevated genital infection risk represents a class-effect adverse reaction of SGLT2i. In the present study, infection risks in both low- and high-dose groups were significantly higher than those in the DPP-4i group, with no clear dose-gradient differences in risk ratios. The pathological mechanism underlying this adverse reaction is well established: SGLT2i increase urinary glucose concentration, leading to elevated glucose exposure of the local genital mucosal surfaces, thereby creating a favorable environment for fungal proliferation and consequently inducing genital fungal infections (25). DPP-4i do not affect renal glucose reabsorption or urinary glucose levels; therefore, they are associated with lower genital infection risk and better overall tolerability (26).

## Limitations

First, this study included only publicly available English-language literature, without retrieving or incorporating studies published in Chinese or other languages, nor covering unpublished gray literature or conference abstracts. This introduces a potential language bias risk. Second, heterogeneity among studies for certain outcomes could not be fully explained. Although dose stratification, subgroup analyses, and sensitivity analyses identified some sources of heterogeneity, the interference of confounding factors—including baseline glycemic levels, diabetes duration, and background therapy regimens—could not be further excluded due to limitations in publicly available data. Third, the follow-up durations of included studies were generally short, predominantly ranging from 12 to 52 weeks. Consequently, these findings reflect only short-term glycemic efficacy, weight-loss effects, and common adverse reactions of the two drug classes, without being able to assess the impact of long-term therapy on cardiorenal hard endpoints or the progression of chronic complications. Fourth, the number of studies and sample sizes in certain subgroups were limited; for example, the ertugliflozin subgroup included only a single study, resulting in insufficient statistical power to fully validate efficacy differences across different SGLT2i agents. Fifth, this study was based solely on published aggregate-level data and did not obtain individual patient-level data, precluding more refined covariate adjustment and stratified analyses, as well as more rigorous handling of missing data. Sixth, meta-regression was not performed to evaluate the impact of continuous covariates such as baseline HbA1c and diabetes duration, as the limited number of studies in each dose stratum precluded reliable estimation. Future studies with larger numbers of trials are warranted to further investigate these potential sources of heterogeneity.

## Implications for practice, policy, and future research

In clinical practice, individualized drug selection can be guided by patient glycemic control targets, weight management requirements, and tolerance to infection risk. For patients requiring intensive glycemic control or those with overweight/obesity, high-dose SGLT2i may be considered, accompanied by appropriate risk warning and health education regarding genital tract infections. Relevant health policy and clinical practice guideline development may incorporate the efficacy and safety profiles of the two drug classes to optimize clinical treatment pathways for glucose-lowering agents. Future studies should conduct RCTs with longer follow-up durations to supplement evidence regarding the effects of these two drug classes on cardiorenal hard endpoints and long-term complications. Additionally, expanding the literature search scope to include multilingual and multiregional studies would help reduce language bias. Further refinement of efficacy differences across different drug types and dosage regimens would provide more comprehensive and high-quality evidence to support precise clinical medication decisions.

## Conclusions

This meta-analysis demonstrated that, in patients with type 2 diabetes mellitus, low-dose sodium-glucose linked transporter 2 inhibitors achieved HbA1c control comparable to that of dipeptidyl peptidase 4 inhibitors, whereas high-dose sodium-glucose linked transporter 2 inhibitors were significantly superior to dipeptidyl peptidase 4 inhibitors in glycemic efficacy. Both low- and high-dose sodium-glucose linked transporter 2 inhibitors were associated with significantly greater weight reduction compared with dipeptidyl peptidase 4 inhibitors, but with a higher risk of genital infections. These findings provide evidence-based reference for individualized glucose-lowering agent selection in patients with type 2 diabetes mellitus. Clinical application should integrate comprehensive considerations of patient glycemic control requirements, weight management goals, and infection risk.

## Data Availability

The original contributions presented in the study are included in the article/**Supplementary Material**. Further inquiries can be directed to the corresponding author.
