# Supplementary material for: SGLT2 versus DPP-4 inhibitors in type 2 diabetes: a meta-analysis of outcomes
Source: Front Endocrinol (Lausanne). 2026 Sep 4;17:1930813. doi: 10.3389/fendo.2026.1930813 (PMC13585633; doi:10.3389/fendo.2026.1930813)
Supplement: Supplementary file 1 [file SupplementaryFile1.docx]

**Electronic Supplementary Material**

**Table S1.** Systematic literature review search terms and strategy.

**Search terms for PubMed**

#1 type 2 diabetes mellitus[Title/Abstract] OR T2DM[Title/Abstract]

#2 sodium-glucose transporter 2 inhibitors[Title/Abstract] OR SGLT2 inhibitors[Title/Abstract] OR SGLT2i[Title/Abstract]

#3 dipeptidyl peptidase 4 inhibitors[Title/Abstract] OR DPP-4 inhibitors[Title/Abstract] OR DPP4i[Title/Abstract]

#4 randomized controlled trial[pt] OR RCT[Title/Abstract] OR randomized[Title/Abstract]

#5 #1 AND #2 AND #3 AND #4

**Search terms for Embase**

#1 'type 2 diabetes mellitus'/exp OR T2DM.mp.

#2 'sodium-glucose transporter 2 inhibitors'/exp OR 'SGLT2 inhibitors'/exp OR SGLT2i.mp.

#3 'dipeptidyl peptidase 4 inhibitors'/exp OR 'DPP-4 inhibitors'/exp OR DPP4i.mp.

#4 'randomized controlled trial'/exp OR RCT.mp. OR randomized.mp.

#5 #1 AND #2 AND #3 AND #4

**Search terms for Web of Science**

TS=(type 2 diabetes mellitus OR T2DM) AND TS=(sodium-glucose transporter 2 inhibitors OR SGLT2 inhibitors OR SGLT2i) AND TS=(dipeptidyl peptidase 4 inhibitors OR DPP-4 inhibitors OR DPP4i) AND TS=(Randomized Controlled Trial OR RCT OR randomized)

**Search terms for Cochrane Library**

MeSH Terms: Type 2 Diabetes Mellitus, Sodium-Glucose Transporter 2 Inhibitors, Dipeptidyl Peptidase 4 Inhibitors, Randomized Controlled TrialKey Words: T2DM, SGLT2 inhibitors, SGLT2i, DPP-4 inhibitors, DPP4i, RCT, randomized controlled trialCombination: (type 2 diabetes mellitus OR T2DM) AND (sodium-glucose transporter 2 inhibitors OR SGLT2 inhibitors OR SGLT2i) AND (dipeptidyl peptidase 4 inhibitors OR DPP-4 inhibitors OR DPP4i) AND (Randomized Controlled Trial OR RCT)

**Table S2.** Baseline characteristics included in the study

| First Author and Year | sample size | | Age: Years | | Gender: Male or Female | | BMI（kg/m²） | | HbA1c（%） | | FPG（mmol/L or mg/dL） | | eGFR（mL/min/1.73m²） | | blood fat （TC/HDL/LDL/TG） | | Diabetes disease duration (years) | | SBP/DBP（mmHg） | |
| --- | --- | --- | --- | --- | --- | --- | --- | --- | --- | --- | --- | --- | --- | --- | --- | --- | --- | --- | --- | --- |
|  | SGLT2i | DPP-4i | SGLT2i | DPP-4i | SGLT2i | DPP-4i | SGLT2i | DPP-4i | SGLT2i | DPP-4i | SGLT2i | DPP-4i | SGLT2i | DPP-4i | SGLT2i | DPP-4i | SGLT2i | DPP-4i | SGLT2i | DPP-4i |
| DeFronzo 2015 | 277 | 128 | 55.8 ± 10.2 | 56.2 ± 10.0 | 143 / 134 | 64 / 64 | 31.4 ± 5.4 | 30.6 ± 5.4 | 8.01 ± 0.88 | 8.02 ± 0.90 | 160.7 ± 36.2 | 156.3 ± 30.7 | 90.7 ± 18.4 | 90.0 ± 20.1 | NR | NR | NR | NR | 130.4 / 80.1 | 128.4 / 77.7 |
| Laffel 2023 | 52 | 52 | 14.5 ± 1.9 | 14.9 ± 1.8 | NR | NR | 34.4 ± 6.8 | 34.0 ± 6.5 | 8.00 ± 1.29 | 8.05 ± 1.11 | 154.43 ± 57.78 | 162.81 ± 56.01 | 126.1 ± 30.0 | 125.1 ± 25.4 | NR | NR | 1.4 ± 1.2 | 1.5 ± 1.1 | 116.0 ± 10.0 / 69.4 ± 8.3 | 113.6 ± 9.3 / 69.4 ± 7.9 |
| Lavalle-González 2013 | 735 | 366 | 55.4 ± 9.3 | 55.5 ± 9.6 | 339 / 396 | 172 / 194 | 31.9 ± 6.3 | 32.0 ± 6.1 | 7.9 ± 0.9 | 7.9 ± 0.9 | 9.45 ± 2.4 | 9.4 ± 2.3 | NR | NR | NR | NR | 6.9 ± 5.4 | 6.8 ± 5.2 | NR | NR |
| Lewin 2015 | 267 | 133 | 55.0 ± 9.8 | 53.8 ± 11.5 | 141 / 124 | 75 / 58 | 31.35 ± 5.7 | 31.9 ± 5.9 | 8.02 ± 1.00 | 8.05 ± 0.89 | 156.5 ± 40.3 | 156.0 ± 37.1 | 88.45 ± 18.6 | 89.5 ± 20.3 | NR | NR | NR | NR | 129.1 / 79.0 | 127.7 / 78.2 |
| Pratley 2018 | 498 | 247 | 55.2 ± 9.8 | 54.8 ± 10.7 | 261 / 235 | 154 / 93 | 31.65 ± 6.0 | 31.7 ± 6.5 | 8.6 ± 1.0 | 8.5 ± 1.0 | 181.8 ± 49.0 | 177.4 ± 46.6 | 92.35 ± 20.9 | 92.6 ± 18.2 | NR | NR | 7.2 ± 5.4 | 6.2 ± 5.2 | 129.3 / 77.8 | 128.3 / 77.5 |
| Roden 2013 | 448 | 223 | 55.0 ± 11.2 | 55.1 ± 9.9 | 287 / 161 | 141 / 82 | 28.25 ± 5.5 | 28.2 ± 5.2 | 7.87 ± 0.86 | 7.85 ± 0.79 | 8.47 ± 1.84 | 8.16 ± 1.60 | 87.65 ± 18.7 | 87.6 ± 17.3 | NR | NR | NR | NR | 128.1 / 77.35 | 132.7 / 80.5 |
| Rosenstock 2012 | 193 | 65 | 52.3 ± 7.5 | 51.7 ± 8.1 | 105 / 88 | 38 / 27 | 31.6 ± 5.0 | 31.6 ± 5.0 | 7.71 ± 0.93 | 7.64 ± 0.95 | 162.4 ± 41.1 | 158 ± 42 | NR | NR | NR | NR | 6.1 ± 5.2 | 5.6 ± 4.7 | 125.6 ± 12.0 / 78.3 ± 8.3 | 129 ± 13 / 79 ± 8 |
| Rosenstock 2013 | 141 | 71 | 59.0 ± 8.6 | 58 ± 10.1 | 70 / 71 | 38 / 33 | 31.45 ± 4.4 | 31.0 ± 4.5 | 8.0 ± 0.75 | 8.1 ± 0.9 | 176.5 ± 38.6 | 178 ± 44 | NR | NR | NR | NR | NR | NR | 133.85 / 78.6 | 135.5 / 80.5 |
| Schernthaner 2013 | 377 | 378 | 56.6 ± 9.6 | 56.7 ± 9.3 | 207 / 170 | 215 / 163 | 31.5 ± 6.9 | 31.7 ± 6.9 | 8.1 ± 0.9 | 8.1 ± 0.9 | 9.4 ± 2.6 | 9.2 ± 2.5 | NR | NR | NR | NR | 9.4 ± 6.1 | 9.7 ± 6.3 | NR | NR |
| Scott 2018 | 306 | 307 | 66.6 ± 8.6 | 67.7 ± 8.5 | 186 / 120 | 169 / 138 | 31.5 ± 5.3 | 31.8 ± 5.7 | 7.8 ± 0.7 | 7.7 ± 0.7 | 9.2 ± 2.3 | 9.0 ± 2.2 | 76.9 ± 12.3 | 79.4 ± 11.3 | NR | NR | 10.7 ± 7.4 | 10.5 ± 7.0 | NR | NR |

**Note:** NR, not reported; SGLT2i, sodium-glucose linked transporter 2 inhibitors; DPP-4i, dipeptidyl peptidase 4 inhibitors; BMI, body mass index; HbA1c, glycated hemoglobin A1c; FPG, fasting plasma glucose; eGFR, estimated glomerular filtration rate; TC, total cholesterol; HDL, high-density lipoprotein cholesterol; LDL, low-density lipoprotein cholesterol; TG, triglycerides; SBP, systolic blood pressure; DBP, diastolic blood pressure.

**Table S3.** Results of Egger’s Test.

| Outcome Index | β₁ | SE | Z | P |
| --- | --- | --- | --- | --- |
| HbA1c (low-dose SGLT2i) | -1.35 | 0.893 | -1.51 | 0.131 |
| HbA1c (high-dose SGLT2i) | 1.25 | 1.911 | 0.65 | 0.513 |
| Body weight (low-dose SGLT2i) | 0.16 | 1.412 | 0.11 | 0.911 |
| Body weight (high-dose SGLT2i) | 1.64 | 3.459 | 0.47 | 0.635 |
| Genital infection (low-dose SGLT2i) | 0.24 | 1.171 | 0.2 | 0.84 |
| Genital infection (high-dose SGLT2i) | -1.51 | 0.935 | -1.62 | 0.106 |

**Note:** β₁, regression coefficient; SE, standard error; HbA1c, glycated hemoglobin A1c; SGLT2i, sodium-glucose linked transporter 2 inhibitors. *P* > 0.05 indicates no significant publication bias.

**Table S4.** GRADE evidence quality assessment

| Outcome | Risk of bias | Inconsistency | Indirectness | Imprecision | Publication bias | Quality of evidence |
| --- | --- | --- | --- | --- | --- | --- |
| HbA1c (low-dose SGLT2i) | No serious downgrade | Downgrade 1 level | No serious downgrade | No serious downgrade | No serious downgrade | Moderate |
| HbA1c (high-dose SGLT2i) | No serious downgrade | Downgrade 1 level | No serious downgrade | No serious downgrade | No serious downgrade | Moderate |
| Weight (low-dose SGLT2i) | No serious downgrade | No serious downgrade | No serious downgrade | No serious downgrade | No serious downgrade | High |
| Weight (high-dose SGLT2i) | No serious downgrade | Downgrade 1 level | No serious downgrade | No serious downgrade | No serious downgrade | Moderate |
| Genital infection (low-dose SGLT2i) | No serious downgrade | No serious downgrade | No serious downgrade | Downgrade 1 level | No serious downgrade | Moderate |
| Genital infection (high-dose SGLT2i) | No serious downgrade | No serious downgrade | No serious downgrade | Downgrade 1 level | No serious downgrade | Moderate |

**Note:** GRADE, Grading of Recommendations Assessment, Development and Evaluation; HbA1c, glycated hemoglobin A1c; SGLT2i, sodium-glucose linked transporter 2 inhibitors

**Figure S1.** Flow chart of included literature.

**Figure S2.** Results of bias risk in RCT literature.

**Figure S3.** Funnel Chart.
